# Hsp90 inhibition leads to an increase in surface expression of multiple immunological receptors in cancer cells

**DOI:** 10.3389/fmolb.2024.1334876

**Published:** 2024-04-05

**Authors:** Madison Wickenberg, Rebecca Mercier, Megan Yap, John Walker, Kristi Baker, Paul LaPointe

**Affiliations:** ^1^ Department of Cell Biology, Faculty of Medicine and Dentistry, University of Alberta, Edmonton, AB, Canada; ^2^ Department of Oncology, Faculty of Medicine and Dentistry, University of Alberta, Edmonton, AB, Canada

**Keywords:** cancer, chaperones, proteostasis, immunological receptors, HSP90 (heat shock protein 90)

## Abstract

Heat shock protein 90 (Hsp90) is a molecular chaperone important for maintaining protein homeostasis (proteostasis) in the cell. Hsp90 inhibitors are being explored as cancer therapeutics because of their ability to disrupt proteostasis. Inhibiting Hsp90 increases surface density of the immunological receptor Major Histocompatibility Complex 1 (MHC1). Here we show that this increase occurs across multiple cancer cell lines and with both cytosol-specific and pan-Hsp90 inhibitors. We demonstrate that Hsp90 inhibition also alters surface expression of both IFNGR and PD-L1, two additional immunological receptors that play a significant role in anti-tumour or anti-immune activity in the tumour microenvironment. Hsp90 also negatively regulates IFN-γ activity in cancer cells, suggesting it has a unique role in mediating the immune system’s response to cancer. Our data suggests a strong link between Hsp90 activity and the pathways that govern anti-tumour immunity. This highlights the potential for the use of an Hsp90 inhibitor in combination with another currently available cancer treatment, immune checkpoint blockade therapy, which works to prevent immune evasion of cancer cells. Combination checkpoint inhibitor therapy and the use of an Hsp90 inhibitor may potentiate the therapeutic benefits of both treatments and improve prognosis for cancer patients.

## Introduction

Different forms of immunotherapy are currently available for cancer treatment such as the use of viral therapy, immune checkpoint inhibitor therapy (ICIT), monoclonal antibodies, and adoptive cell therapy (Zhang and Zhang, 2020). ICITemploys neutralizing monoclonal antibodies that target critical immune signaling molecules such as the cytotoxic T-lymphocyte-associated protein 4 (CTLA-4), programmed cell death protein 1 (PD-1), or programmed death-ligand 1 (PD-L1) (Farkona et al., 2016; Seidel et al., 2018; Liu et al., 2021). Anti-CTLA-4 immunotherapy works by blocking CTLA-4 which is constitutively expressed by Treg cells (Seidel et al., 2018). CTLA-4 has an inhibitory effect on T-cell function as it prevents CD28 signaling. Inhibiting CTLA-4 leads to increased T-cell activation and more effective immune recognition of tumours. Anti-PD-1/PD-L1 immunotherapy works by blocking either PD-1 or PD-L1 (Akinleye and Rasool, 2019). Cancer cells that have acquired the ability to express PD-L1 can interact with PD-1 found on T-cells and suppress T-cell-mediated killing (Liu et al., 2021). Neutralizing the PD1/PD-L1 interaction restores immune cell function and leads to tumour cell killing. Recently, immunotherapy has become the standard-of-care for a significant group of cancer patients and has been shown to significantly prolong survival in patients with various metastatic tumours better than chemotherapy (Nixon et al., 2018). Additionally, targeting multiple immune modulators simultaneously is more effective in preventing disease progression than individually (Hodi et al., 2016; Wolchok et al., 2017; He and Xu, 2020). Despite these advances, not all patients respond to these therapies and the mechanisms that govern their immune resistance are being intensely investigated.

Identifying cancer treatments that synergize with ICIT is an important objective of current research. Recent studies have shown that a competent immune system is necessary for chemotherapy to be effective (Merlano et al., 2022). Therefore, it is no surprise that combining immunotherapy, a treatment that boosts immune recognition, with traditional chemotherapy is more effective than chemotherapy alone (Paz-Ares et al., 2018; Leonetti et al., 2019; Xiao et al., 2022). This is likely due to increased presentation of tumour neoantigens that are released upon chemotherapy-induced death of the cancer cells.

There are other facets of the tumour microenvironment that may modify anti-tumour immune responses. One such key regulator is interferon gamma (IFN-γ), which is produced by infiltrating immune cells. The action of IFN-γ requires the expression the IFN-γ receptor (IFNGR) on the cell surface to trigger the activation of intracellular mediators such as STAT1 which in turn activates the transcription of immunomodulatory genes (Jorgovanovic et al., 2020). Strikingly, IFN-γ has both protumour and antitumour effects within the tumour microenvironment (Zaidi, 2019; Gocher et al., 2022).

One well-understood role of IFN-γ is that it triggers upregulation of the Major Histocompatibility Complex 1 (MHC1) receptor on the cell surface; this receptor provides information to the immune system regarding the cell’s origin and improves T cell recognition of infected or damaged cells (Zhou, 2009; Dhatchinamoorthy et al., 2021). This receptor is found on the surface of all nucleated cells where it forms complexes with internally derived peptides and presents these to T cells (Wieczorek et al., 2017). The peptides are generated by proteasomal degradation of proteins in cells as a result of general protein turnover or due to protein misfolding. The peptides are loaded onto MHC1 receptor complexes in the ER and then are trafficked to the cell surface where T cells can monitor the internal composition of the cell through detection of these peptides (Sari and Rock, 2023). If the peptides being presented contain mutated amino acid sequences, which occurs in the case of cancer, T-cells can detect that the sequences are unique and therefore that the cells are foreign (Wieczorek et al., 2017). Not surprisingly, downregulation of MHC1 in patients is correlated with poor prognosis as it leads to decreased T-cell recognition (Watson et al., 2006; Cornel et al., 2020). IFN-γ thus helps to combat this immune evasion strategy. However, IFN-γ can also increase the expression of PD-L1 in cancer cells to escape immunosurveillance (Cha et al., 2019; Hoekstra et al., 2020). Importantly, while the upregulation of PD-L1 by IFN-γ could negate the benefit of increasing MHC1, this problem can be overcome by anti-PD1/PD-L1 therapy (Abiko et al., 2015; Gocher et al., 2022).

A particularly important characteristic of cancer cells is that they contain highly mutated and thermodynamically unstable proteins and as a result rely heavily on mechanisms to ensure proper protein folding and prevent degradation (Miyata et al., 2013). Therefore, exploiting the cell’s reliance on these mechanisms is an important area of research for cancer treatment. One example of a mechanism that cancer cells rely on is the presence of molecular chaperones, a class of proteins that interact with nascent or misfolded proteins to assist in proper protein folding (Zhang et al., 2015). Hsp90 is a molecular chaperone and is of particular importance as it has been shown to be a critical component of cells that are exposed to proteostasis stressors such as in neurodegenerative diseases or cancer (Whitesell and Lindquist, 2005; Luo et al., 2010). Additionally, the multiple paralogues of Hsp90 have all been shown to have specific roles in tumour progression (Albakova et al., 2022). One potential way to enhance the immunogenicity of a cancer cell is through the use of an Hsp90 inhibitor (Jaeger et al., 2019; Zavareh et al., 2021; Rahmy et al., 2022). Hsp90 inhibition leads to decreased tumour mass in a mouse model, but only in mice with a competent immune system. This suggests that not only is Hsp90 a potential target for cancer treatment because mutated oncoproteins often depend on this chaperone for their activity, there is also an important interplay between Hsp90 inhibition and the immune system (Grbovic et al., 2006; Luo et al., 2017). This has prompted us to investigate how proteostasis, and the disruption of proteostasis through Hsp90 inhibition, impacts specific components of immunogenicity.

## Results

### Hsp90 inhibition increases tumour neoantigen-loaded MHC1 expression on the surface of mouse colon cancer cells

To test the functional consequence of Hsp90 inhibition on anti-tumour immunity, we used the MC38 mouse colorectal cancer (CRC) model with variants made to resemble two clinically relevant subtypes. The deletion of Mlh1 in MC38 cells results in a high mutational burden and resembles the microsatellite instability (MSI) CRC phenotype and overexpression of KRAS in MC38 cells drives a phenotype that resembles the chromosomal instability (CIN) phenotype (Mowat et al., 2021). These cells also express the ovalbumin protein which harbours the SIINFEKL peptide. Since this peptide is not normally expressed in mammalian cells, it acts as a surrogate tumour neoantigen. When the SIINFEKL peptide is loaded onto MHC1 complexes (known as H2-K^b^ in mice), it can be recognized at the cell surface by CD8^+^ T-cells from OT1 T-cell transgenic mice that express a SIINFEKL-specific T cell receptor (Hogquist et al., 1994). Co-culturing OT1 T cells with MC38 cells that were pretreated or not with the Hsp90 inhibitor NVP-AUY922 thus provides an opportunity to directly measure the impact of Hsp90 inhibition on activation of tumour-specific T cells. These CRC subtypes are typically associated with different mutational burdens so we wondered if they may respond differently to Hsp90 inhibition.

**FIGURE 1:**
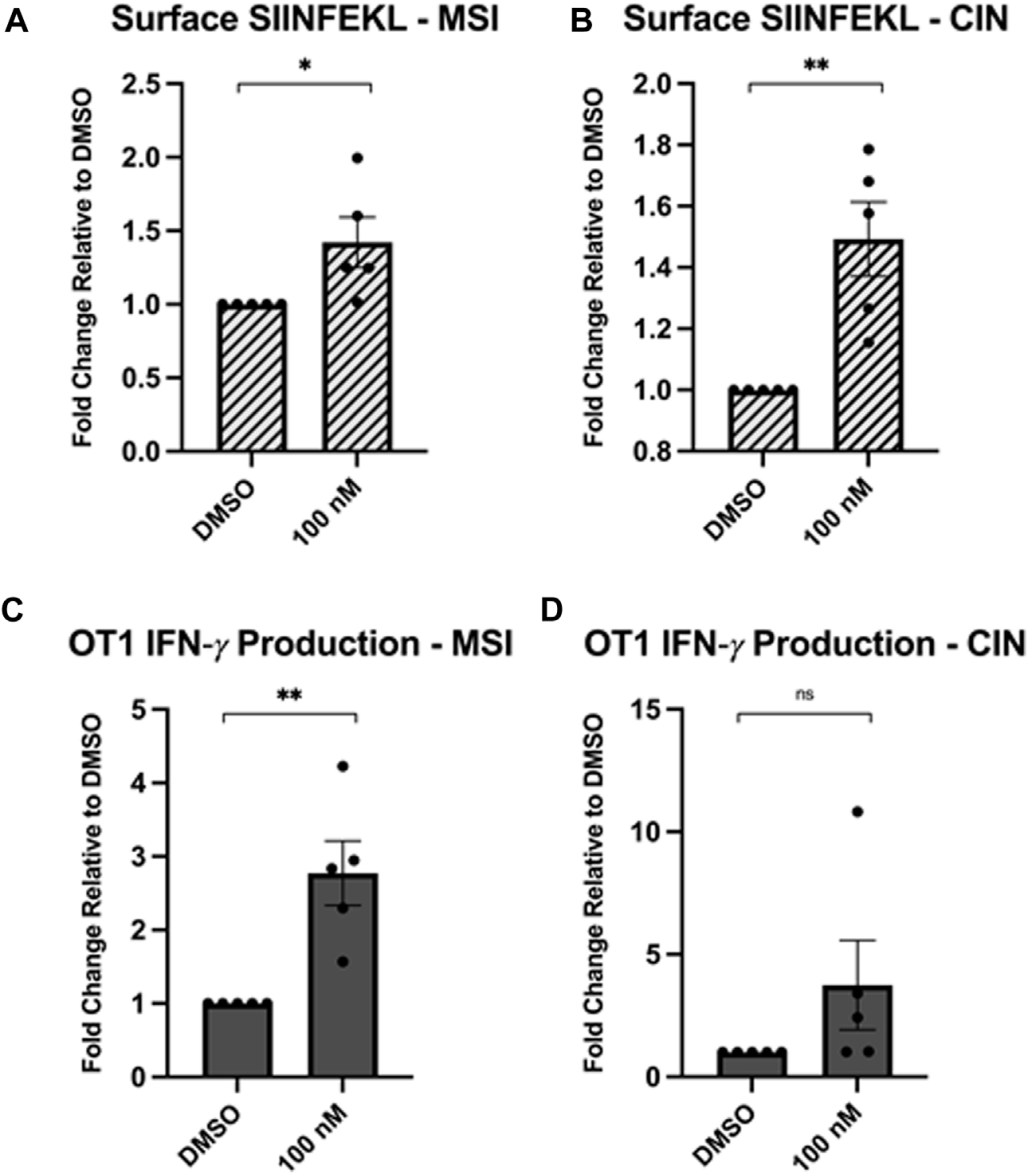
Hsp90 inhibition increases neoantigen-specific immunogenicity of cancer cells. MC38 cells (mouse colorectal cancer) lacking the *Mlh1* gene, known as microsatellite instability (MSI) cells, or with a mutated Kras gene, known as chromosomal instability (CIN) cells that overexpress the OVA-derived SIINFEKL neoantigen were treated with 0.1 μM NVPAUY922 for 24 h. The inhibitors were then removed and SIINFEKL-specific OTI CD8^+^ T cells were added and cocultured with the cancer cells for 48 h. **(A,B)** Expression of the H2-K^b^-bound SIINFEKL epitope was analyzed on either the MSI or CIN cell lines using flow cytometry. **(C,D)** Intracellular expression of IFN-γ was assessed in SIINFEKL-specific CD8^+^ T cells using flow cytometry. Experiments were carried out three times in triplicate (n = 3) and statistical significance was determined using a *t*-test comparing the mean between the two treatment groups (* denotes *p* < 0.5; ** denotes *p* < 0.01; *** denotes *p* < 0.001; **** denotes *p* < 0.0001). Error bars show standard error of the mean.

We inhibited Hsp90 in MC38 cells with a low concentration of NVP-AUY922 (100 nM) and measured the surface expression of H2-K^b^ complexes loaded with SIINFEKL. We observed an increase in SIINFEKL-loaded H2-K^b^ after Hsp90 inhibition with the drug (Figures 1A, B). We also observed enhanced IFN-γ production by OT1 cells co-cultured with the NVP-AUY922-treated MC38 cells (Figures 1C, D). There was not a statistically significant increase in IFN-γ production by OT1 cells in experiments using CIN cells (Figure 1D), but this is likely due to the high variability in results between experiments. All experiments using showed an increase in IFN-γ production relative to the control group, however the magnitude varied between each experiment. Ultimately, both the changes in SIINFEKL-loaded H2-K^b^ and IFN-γ production by OT1 cells indicated that Hsp90 inhibition in cancer cells can result in higher activation of tumour-specific CD8^+^ T cells.

### Hsp90 inhibition upregulates MHC1 surface expression in multiple cancer cell lines

**FIGURE 2:**
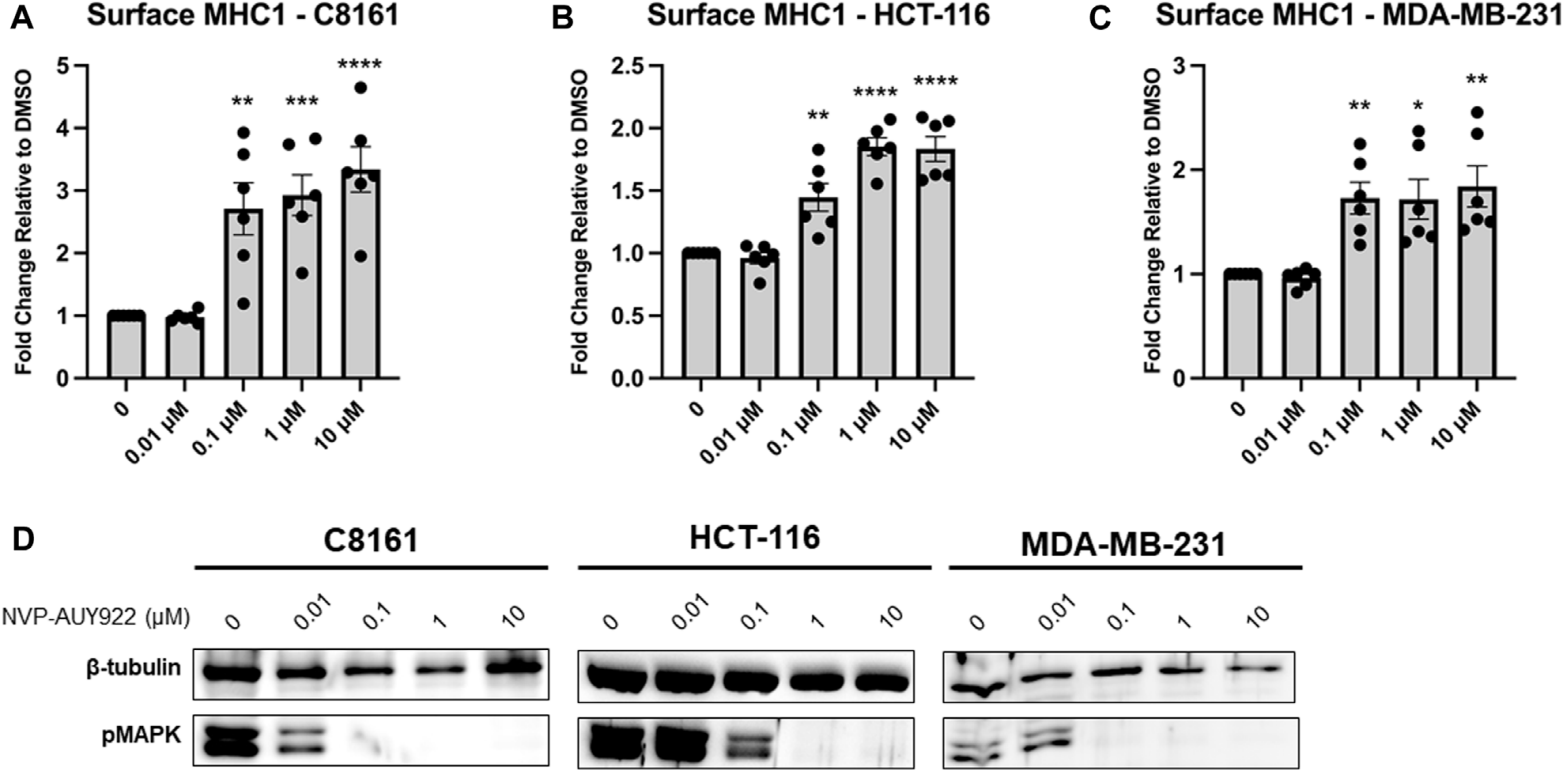
**(A–C)** Inhibition of Hsp90 in C8161, HCT-116, and MDA-MB-231 cells with NVP-AUY922 results in an increase in surface MHC1 complexes. C8161 (melanoma), HCT116 (colorectal carcinoma), and MDA-MB-231 (breast cancer) cells were treated for 48 h with indicated concentrations of NVP-AUY922. The abundance of HLA complexes was measured by flow cytometry using the W6/32 antibody conjugated to PE and expressed as a fold-change over vehicle-treated cells. A dose-dependent increase in MHC1 complex abundance was detected with the inhibitor and in all 3 cell lines. Experiments were carried out six (n = 6) times in triplicate. Statistical significance was determined using a *t*-test comparing the mean between each treatment group and the control group (* denotes *p* < 0.5; ** denotes *p* < 0.01; *** denotes *p* < 0.001; **** denotes *p* < 0.0001). Error bars show standard error of the mean. **(D)** HCT-116 cells treated with indicated concentrations of NVP-AUY922 show reduced protein levels of pMAPK. β-tubulin is used as a loading control.

We next wondered if Hsp90 inhibition would have a similar effect in human cancer cells lines. We chose three cancer cell lines for testing. Melanoma is frequently treated with ICIT so we chose C8161 (melanoma) which was first characterized as a highly invasive and metastatic melanoma cell line (Welch et al., 1991). ICIT is currently being expanded into colorectal cancers (Makaremi et al., 2021) and breast cancers (Thomas et al., 2020) so we chose two cancer cell lines, HCT-116 (colorectal cancer), and MDA-MB-231 (triple-negative breast cancer), that also have a relatively high mutational burden (Ghandi et al., 2019) [which has been shown to be an important indicator for response (Klempner et al., 2020; Aggarwal et al., 2023)]. We tested each cell line with increasing concentrations of NVP-AUY922 and measured MHC1 at the cell surface via flow cytometry. We observed a dose-dependent increase in MHC1 surface density in C8161, HCT-116, and MDA-MB-231 cells treated with NVP-AUY922 (Figures 2A–C). To confirm that our treatment was effectively inhibiting Hsp90, we stained for phosphorylated MAPK which is a hallmark of Hsp90 inhibition since MEK, the kinase responsible for MAPK phosphorylation, is a client of Hsp90. We detected a decrease in levels of phosphorylated MAPK at concentrations of NVP-AUY922 that resulted in an increase in surface MHC1 staining (Figure 2D).

### Inhibition of cytosolic Hsp90 is sufficient for MHC1 upregulation

**FIGURE 3:**
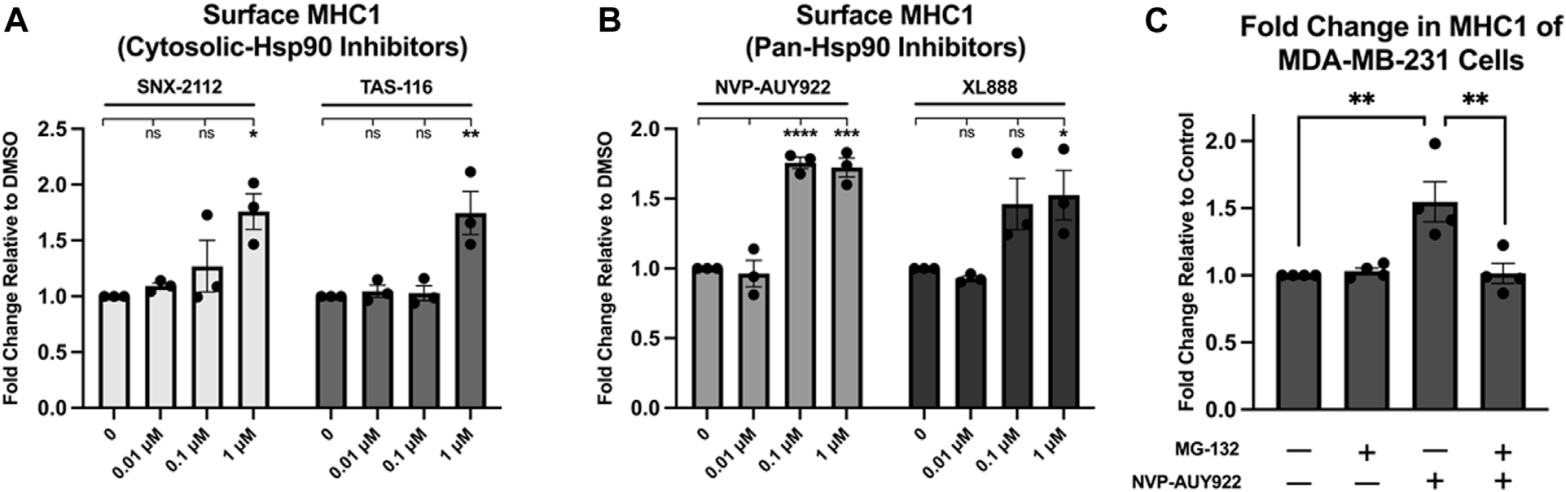
**(A,B)** Inhibition of Hsp90 in MDA-MB-231 cells using cytosolic inhibitors results in a fold increase in MHC1 density similar to that of cells treated with a pan-Hsp90 inhibitor. Cells were treated with increasing concentrations of either pan or cytosolic inhibitor for 48 h. The abundance of HLA complexes was measured by flow cytometry using the W6/32 antibody conjugated to PE and expressed as a fold-change over vehicle-treated cells. A dose-dependent increase in MHC1 complex abundance was detected with all four inhibitors. **(C)** Inhibition of the proteasome dissipates any increase in MHC1 seen with Hsp90 inhibition. MDA-MB-231 cells were treated with either vehicle, 1 µM NVP-AUY922, 1 µM of proteasome inhibitor MG-132, or both for 24H. The abundance of HLA complexes was measured by flow cytometry using the W6/32 antibody conjugated to PE and expressed as a fold-change over vehicle-treated cells. An increase in MHC1 complexes was observed in cells treated with NVP-AUY922, but not in cells treated with both NVP-AUY922 and MG-132. Experiments were carried out three (n = 3) times in triplicate. Statistical significance was determined using a Tukey’s multiple comparison test between each treated sample (* denotes *p* < 0.5; ** denotes *p* < 0.01; *** denotes *p* < 0.001; **** denotes *p* < 0.0001). Error bars show standard error of the mean.

NVP-AUY922 targets the ATP-binding site of all four Hsp90 paralogues (*i.e*., Hsp90ɑ, Hsp90β, TRAP1, GRP94). The paralogues TRAP1 and GRP94 are located in the mitochondria and endoplasmic reticulum respectively, while Hsp90ɑ and Hsp90β are found in the cytosol (Hoter et al., 2018; Mercier and LaPointe, 2022). To determine if inhibition of the cytosolic paralogues alone would be sufficient for increasing MHC1 surface presentation, we tested two cytosol-specific Hsp90 inhibitors along with the pan-Hsp90 inhibitor NVP-AUY922 and an additional pan-Hsp90 inhibitor. We found that treatment with the two cytosol-specific inhibitors, SNX-2112 and TAS-116, resulted in a similar increase in MHC1 surface expression as that of the two pan-Hsp90 inhibitors NVP-AUY922 and XL888 (Figures 3A, B). Treatment of MDA-MB-231 cells with the proteasome inhibitor, MG-132, reversed the increase in surface MHC1 associated with Hsp90 inhibition with NVP-AUY922 (Figure 3C). This suggested that protein degradation by the proteasome is required for the increase in surface MHC1 after Hsp90 inhibition.

### Hsp90 inhibition affects HLA subtypes differently

**FIGURE 4:**
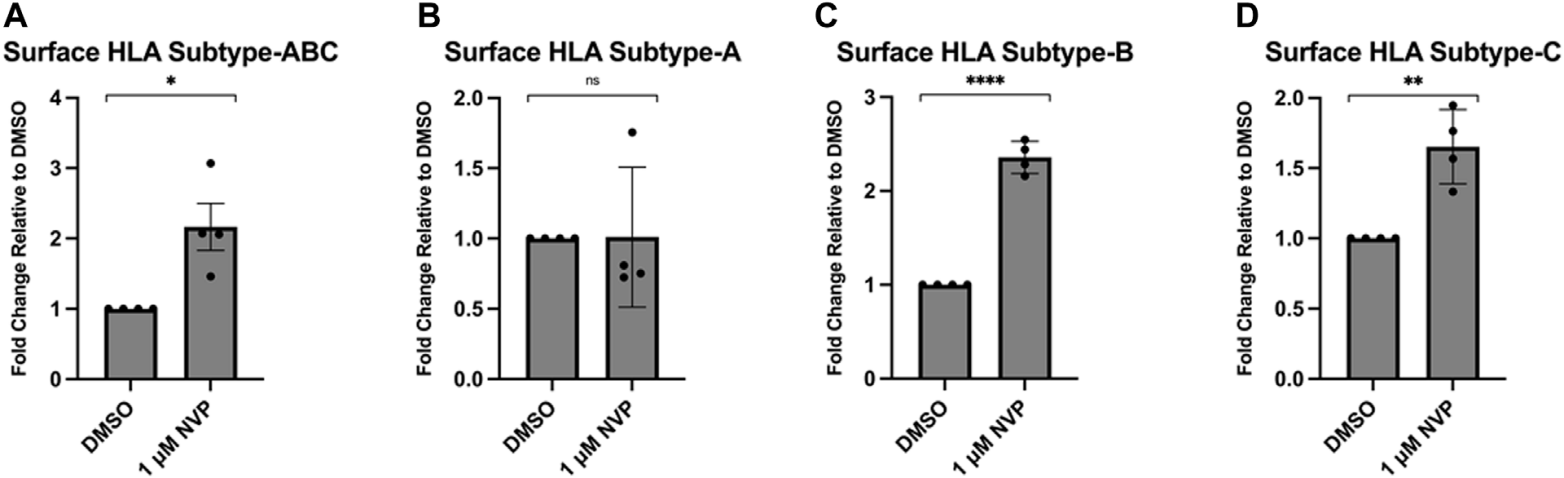
Inhibition of Hsp90 in MDA-MB-231 results in an allelic variant-specific increase in surface HLA complexes. MDA-MB-231 were treated for 48 h with 1 μM NVP-AUY922 **(A–D)**. The abundance of pan-HLA complexes was measured by flow cytometry using the W6/32 antibody conjugated to PE and expressed as a fold-change over vehicle-treated cells **(A)**. Levels of HLA-A **(B)**, HLA-B **(C)** and HLA-C **(D)** were measured via flow cytometry with variant-specific antibodies. Levels of HLA-B and HLA-C, but not HLA-A, increased after treatment with the drug. Experiments were carried out four times in triplicate (n = 4) and statistical significance was determined using a using a *t*-test comparing the mean between the treatment and control groups (* denotes *p* < 0.5; ** denotes *p* < 0.01; *** denotes *p* < 0.001; **** denotes *p* < 0.0001). Error bars show standard error of the mean.

MHC1 complexes are comprised of an invariant β2-microglobulin subunit and one of multiple subtypes of an α-chain. There are at least eight MHC1 α-chains (known as HLAs) (designated A-G) encoded in the human genome. There are three classical MHC1 subtypes (HLA-A, B, and C) and several non-canonical variants (HLA- E, F, and G). The classical HLA subtypes expressed in MDA-MB-231 have been analyzed as well as the peptides that are presented in these HLA complexes (Scholtalbers et al., 2015; Rozanov et al., 2018). Thus, we treated MDA-MB-231 cells with pan-Hsp90 inhibitor NVP-AUY922 (Figures 4A–D) and measured the surface levels of the three classical HLA subtypes (A, B, and C). We observed subtype-specific changes in surface MHC1 levels that occurred with the use of either inhibitor. Surprisingly, we observed an increase in HLA-B and HLA-C MHC1 subtypes and no change in the HLA-A subtype. This suggested either that the loading of peptides (or their post-processed products) occurred preferentially on HLA-B and HLA-C or that the biogenesis of these complexes is regulated by additional pathways unrelated to peptide supply.

### Hsp90 inhibition modulates the expression of other important immunological receptors

**FIGURE 5:**
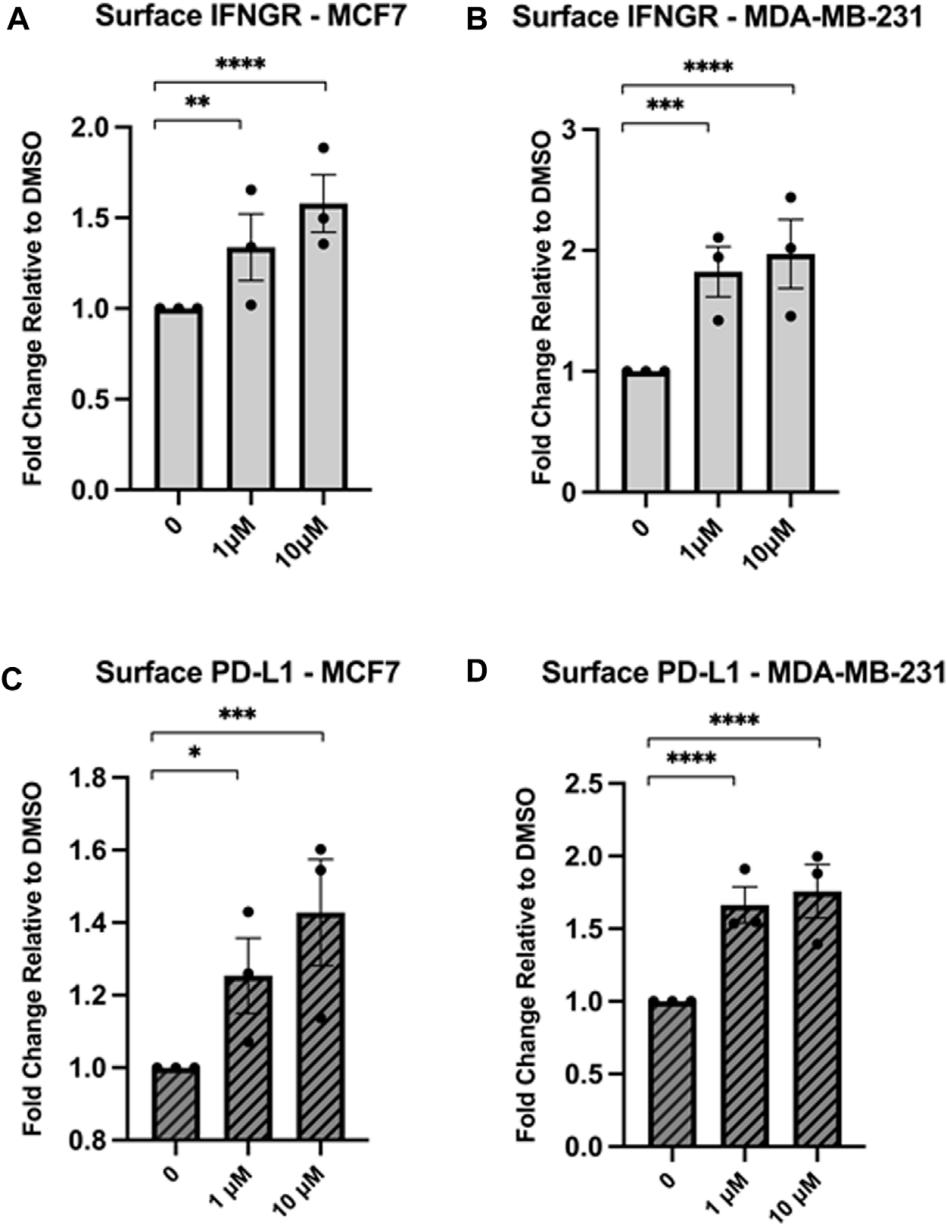
Inhibition of Hsp90 in MCF7 or MDA-MB-231 cell lines results in an increase in the immunological receptors IFNGR and PD-L1. MCF7 or MDA-MB-231 cells were treated for 24 h with NVPAUY922. The abundance of IFNGR **(A,B)** or PD-L1 **(C,D)** was measured using flow cytometry and either a PE-conjugated CD119 antibody to detect IFNGR, or a PE-conjugated PD-L1 antibody that is extracellular domain specific. Levels of IFNGR and PD-L1 increased after treatment with Hsp90 inhibitor in both cell lines. Experiments were carried out three times in triplicate (n = 3) and statistical significance was determined using a *t*-test comparing the mean between each treatment group and the control group (* denotes *p* < 0.5; ** denotes *p* < 0.01; *** denotes *p* < 0.001; **** denotes *p* < 0.0001). Error bars show standard error of the mean.

Given that Hsp90 inhibition increases surface expression of MHC1, an important receptor for immune recognition, we next wanted to determine the effect on other immunological receptors. We treated MCF7 and MDA-MB-231 cells with either 1 µM or 10 µM NVP-AUY922 and measured changes in surface expression of IFNGR or PD-L1. We observed an increase in both of these immunological receptors in both cell lines (Figures 5A–D). Increased IFNGR through Hsp90 inhibition suggests that simultaneous treatment with both an Hsp90 inhibitor and IFN-𝛾 may have a synergistic effect on MHC1 expression and could have beneficial anti-tumour effects. Increased surface PD-L1 induction by Hsp90 inhibition highlights the importance of combinatorial immunotherapies since a PD-L1 neutralizing antibody would mitigate such unwanted anti-immune effects. Since Hsp90 inhibition resulted in an increase in MHCI, PD-L1, and IFNGR, we wondered how surface levels of a known Hsp90 client would be affected. MDA-MB-231 cells express high levels of the Hsp90 client protein, epidermal growth factor receptor (EGFR) (Mueller et al., 2010). Both mutant and wildtype forms of EGFR require Hsp90 for their stability. Hsp90 inhibition resulted in a decrease in surface EGFR in MDA-MB-231 cells (Supplementary Figure S1) suggesting that the effects we observed on MHC1, PD-L1, and IFNGR were not due to a general effect on surface membrane proteins.

### Hsp90 inhibition enhances surface expression of MHC1 independent of IFN-γ signaling

**FIGURE 6:**
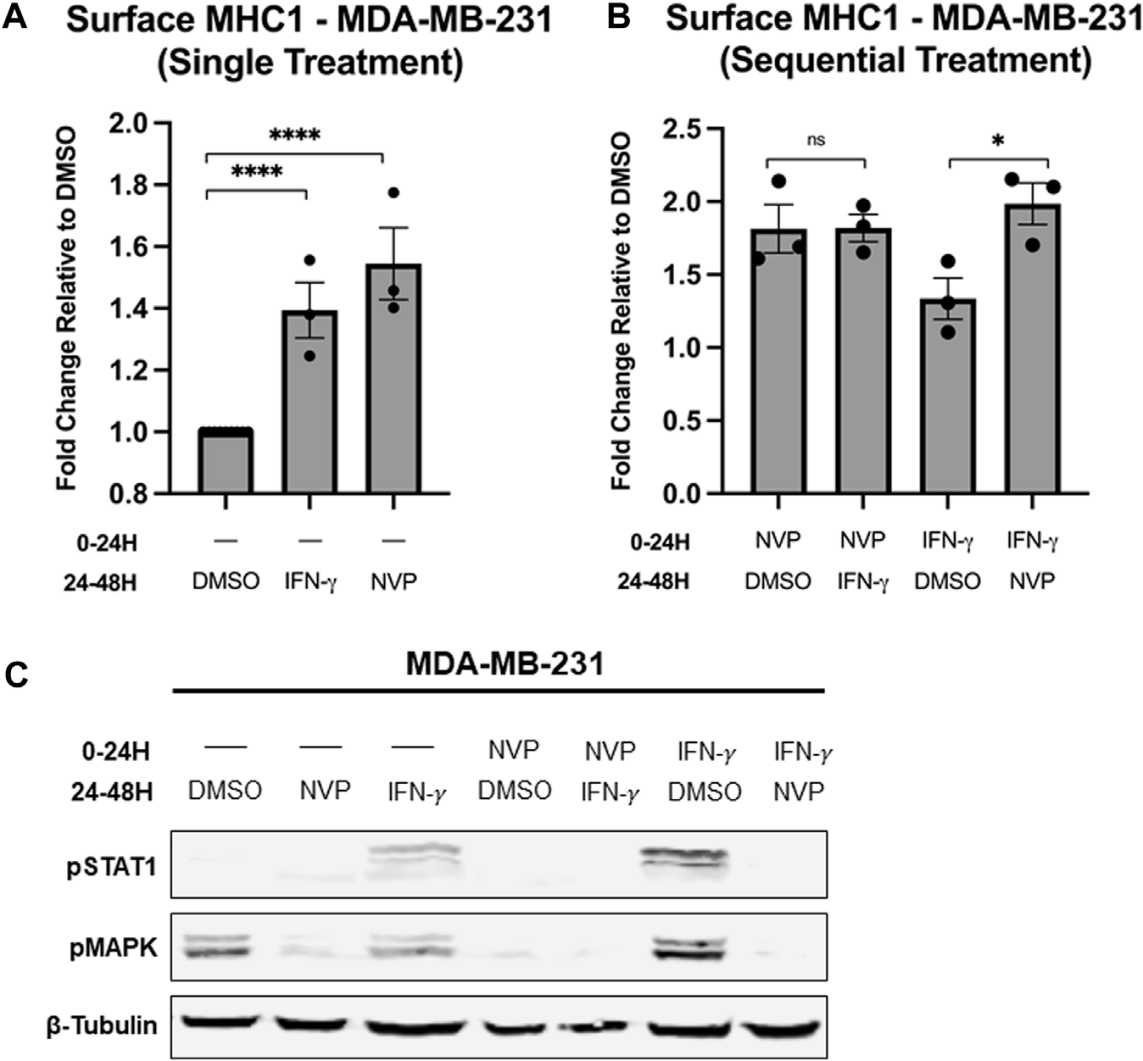
**(A,B)** Treatment of MDA-MB-231 cells with either 100 μg/mL IFN-𝛾 or 10 µM NVP-AUY922 alone results in increased surface MHC1 expression after 24 h. Pre-treatment of MDA-MB-231 cells with NVP-AUY922 before IFN-𝛾 has no statistically significant effect on MHC1 levels. However, pre-treatment with IFN-𝛾 before NVP-AUY922 treatment significantly increases total MHC1 surface expression compared to pre-treatment with DMSO (vehicle). MHC1 complexes were measured by flow cytometry using the W6/32 antibody conjugated to PE and expressed as a fold-change over vehicle-treated cells. Experiments were carried out three times in triplicate (n = 3) and statistical significance was determined using a *t*-test comparing the mean between each treatment group and the control group (* denotes *p* < 0.5; ** denotes *p* < 0.01; *** denotes *p* < 0.001; **** denotes *p* < 0.0001). Error bars show standard error of the mean. **(C)** Treatment of cancer cells with either IFN-𝛾 or NVP-AUY922 has different effects on intracellular protein levels, though both treatments increase MHC1 surface expression. Cell lysates from cells treated as in **(A,B)** were analyzed via Western blot. Cells treated at either the 0–24H timepoint or 24–48H timepoint with NVP-AUY922 have reduced pMAPK protein levels. Cells treated with IFN-𝛾 at either of the two timepoints have increased pSTAT1 protein levels. IFN-𝛾-treated cells either pre-treated or subsequently treated with NVP-AUY922 show no STAT1 phosphorylation. β-tubulin is used as a loading control.

Treatment with either IFN-γ or Hsp90 inhibitor increases MHC1 surface expression and we wanted to determine whether or not this increase occurs through the same pathway (Zhou, 2009). MDA-MB-231 cells either received a single treatment or sequential treatment with two different conditions. The single treatment groups were used as a control to confirm that both IFN-γ and Hsp90 inhibitor increase MHC1 surface expression (Figure 6A). The sequential treatment groups allowed us to determine what overlap exists between the IFN-γ pathway and Hsp90 activity. Interestingly, there was no difference in surface MHC1 levels in cells pre-treated with NVP-AUY922 and then treated with IFN-γ and cells treated only with NVP-AUY922 (Figure 6B). However, cells pre-treated with IFN-γ and then treated with NVP-AUY922 had a significantly higher fold increase in MHC1 surface expression compared to IFN-γ alone (Figure 6B). We used cells treated in parallel to those used during the flow cytometry experiments to examine the changes in levels of proteins via Western blot that are known to be altered by IFN-γ activity or Hsp90 inhibition (Figure 6C). STAT1 is a transcription factor phosphorylated by JAK1/2 following IFN-γ binding to its receptor (Goder et al., 2021). We saw an increase in pSTAT1 levels in cells treated with IFN-γ. However, cells treated with IFN-γ and NVP-AUY922 (in any order) did not show increased pSTAT1 (Figure 6C). Turnover rate of STAT1 could explain why there is no pSTAT1 in cells treated with IFN-γ and then NVP-AUY922, however this would not explain the lack of STAT1 phosphorylation in cells treated with NVP-AUY922 followed by IFN-γ. As in Figure 2, reduction in pMAPK was used as a control for NVP-AUY922 action which occurred in all cells treated with the inhibitor at any timepoint and regardless of if they were also treated with IFN-γ. Ultimately, these results indicate that Hsp90 inhibition negatively regulates IFN-γ activity and the immunological benefits of Hsp90 inhibitors are different than that of IFN-γ signaling, though both result in increased MHC1 surface presentation.

## Materials and methods

### Cell growth and passaging

C8161, HCT-116, MCF7, and MDA-MB-231 cells were grown in 10 cm tissue culture dishes at 37°C with 5% CO2. C8161 and HCT-116 cells were grown in 10 mL Dulbecco’s Modified Eagle Medium (DMEM) (Gibco) supplemented with 10% fetal bovine serum. MCF7 and MDA-MB-231 cells were grown in 10 mL of Roswell Park Memorial Institute (RPMI) 1,640 Medium (Gibco) supplemented with 10% fetal bovine serum. Cells were passaged at 80% confluence or higher by washing with 5 mL of phosphate buffered saline (PBS) (Sigma Aldrich) and then 1 mL of trypsin-EDTA (Gibco) was added to detach the cells from the plate prior to splitting.

All cell lines were obtained from the American Type Culture Collection.

### T-cell activation assays

CD8^+^ T cells were isolated from the spleens and lymph nodes of OT1 mice using the EasySep Mouse CD8^+^ T Cell Isolation Kit (StemCell Technologies). OVA-expressing Mlh1−/− or Kras mutated MC38 mouse CRC cells were first treated for 24 h with 100 nM NVP-AUY922 followed by extensive washing. CD8^+^ T cells were then added at a 5:1 T cell:tumour cell ratio and cultured for 24–48 h. To assess T cell activation, cells were stained for intracellular IFN-γ using the Foxp3/Transcription Factor Staining Buffer Set and anti-IFN-γ-APC (eBioscience). Cells were run on a Cytoflex LS cytometer (Beckman Coulter) and analyzed using FlowJo (BD Bioscience).

### Treatment of cells with NVP-AUY922 or IFN-γ

Hsp90 inhibitor NVP-AUY922 stocks were made using dimethyl sulfoxide (DMSO) as the drug vehicle. The concentration of NVP-AUY922 varied depending on the experiment and all concentrations are listed in the figures. IFN-γ stocks were made by resuspending in water and a final concentration of 100 ng/mL was used for all experiments. Cells were seeded in 12-well dishes at a seeding density of 150,000 cells/well. 1mL of media containing the appropriate treatment was added to each well 24 h after seeding. Cells were then treated for 24 h (unless otherwise specified) before analyzing via either Flow cytometry or Western blot.

### Lysate preparation and western blot analysis

Cells grown in 12-well dishes were washed with 100 µL of phosphate buffered saline (PBS) (Sigma Aldrich). The dishes were kept on ice while 100 µL of 5x sample buffer composed of 10% dithiothreitol and 2% benzonase was added to each well. To prepare samples for running on SDS-Page, the samples were heated to 50°C and spun at 14,000 RPM for 30 s. Approximately 10 μL of sample was added to each lane, using PageRuler Prestained Ladder (ThermoFisher) as the molecular-weight marker. Samples were run on a 10% SDS-PAGE gel and then transferred to a nitrocellulose membrane and analyzed via Western blot. Membranes were blocked in a 5% BSA in TBS-Tween20 solution for 1 h. They were then probed with 1:1,000 dilutions of each primary antibody: β-tubulin anti-rabbit mAb (Cell Signaling 2146S), pMAPK (p44/42) anti-rabbit mAb (Cell Signaling 4695S), or pSTAT (Tyr701) anti-rabbit mAb (Cell Signaling 9167S). Bands were analyzed using either chemiluminescent HRP (Figure 2D) or the LI-COR Odyssey CLx imaging system (Figures 6E, F). For visualization using the HRP system, either a goat anti-mouse (Thermofisher 31430) or a goat anti-rabbit (Thermofisher 31460) IgG secondary antibody was used. For visualization using the LI-COR Odyssey CLx imaging system, either a goat anti-mouse (LI-COR 926-68071) or a goat anti-rabbit (LI-COR 926-32212) IgG secondary antibody was used.

### Flow cytometry analysis

Flow cytometry analysis was done by staining cells with fluorescently-conjugated antibodies to HLA-A2 (Novus Biologicals, NBP1-44896AF488), HLA-B7 (Novus Biologicals, NB100-64159APCCY7), HLA-C (Novus Biologicals, NBP2-50419 PE), a PE-conjugated W6/32 antibody (Santa Cruz Biotechnology, sc-32235) to detect MHC1, a PE-conjugated antibody to detect EGFR1 (Invitrogen, MA5-28544), a PE-conjugated CD119 antibody to detect IFNGR1 (Miltenyi Biotec, 130-125-851), or a PE-conjugated extracellular domain specific PD-L1 antibody (Cell Signaling Technology, 71391). Each sample of cells was obtained from one well of a 12-well dish. The cells were washed using PBS and then stained for 30 min (in darkness at 4°C) using 100 μL of a 1:100 dilution of antibody in FACS buffer solution containing 1%BSA/PBS/0.05% sodium azide. Following staining, cells were washed and then resuspended in 400 μL of FACS buffer solution containing 1%BSA/PBS/0.05% sodium azide. Cells were then visualized using a Fortessa-SORP using gates to exclude for dead cells/debris and doublets (Supplementary Figures S2A, S2B).

### Statistical analysis

Statistical analysis was performed using Prism software (GraphPad). Graphical data was normalized against DMSO and values were calculated as a fold change relative to control. Statistical significance was determined using a *t*-test comparing the mean of the control group to the mean of each treatment group. Error bars depict the Standard Error of the Mean (* denotes *p* < 0.5; ** denotes *p* < 0.01; *** denotes *p* < 0.001; **** denotes *p* < 0.0001).

## Discussion

Hsp90 inhibitors have been explored as anti-cancer agents ever since they were shown to reverse the cellular transformation mediated by v-src (Whitesell et al., 1994). Roughly 20 different Hsp90 inhibitors have been, or are currently, in clinical trials (Li and Luo, 2023) and of these, only one, TAS-116/pimitespib, has gained approval for the treatment of gastrointestinal stromal tumours in Japan (Hoy, 2022; Teranishi et al., 2023a; Teranishi et al., 2023b; Doi et al., 2023). Two naturally occurring Hsp90 inhibitors, geldanamycin and radicicol, we studied for their anti-cancer properties but their use was limited by severe hepatotoxicity (Samuni et al., 2010) and propensity to be derivatized into a biologically inactive form (Agatsuma et al., 2002; Zhou et al., 2010), respectively. However, derivatives of both of these Hsp90 inhibitors have been developed to overcome these limitations. Purine-based Hsp90 inhibitors as well as others developed using a fragment-based approach have been tested in clinical trials (Kim et al., 2009; Neckers and Workman, 2012; Li and Luo, 2023; Magyar et al., 2023). Despite many promising studies, the fact that Hsp90 is so important in other healthy cells may ultimately limit the use of Hsp90 inhibitors as single agent therapies. Still, Hsp90 inhibitors have many attractive qualities that make them amenable to combination therapy. The tumour-retention property of Hsp90 inhibitors (Vilenchik et al., 2004; Banerji et al., 2005) has been explored as a means to image tumours (Barrott et al., 2013; Osada et al., 2022), as well as to deliver larger drug-containing conjugates to tumour cells (Proia et al., 2015; Heske et al., 2016).

The potential of Hsp90 inhibitors to synergize with other therapies is only beginning to be explored but a growing body of evidence suggests that they have the potential to do so with immune checkpoint inhibitor therapy. Hsp90 inhibitors can significantly reduce tumour mass in a mouse model by increasing immune detection and destruction of the tumour cells (Jaeger et al., 2019; Zavareh et al., 2021). Similarly, a paralogue-specific Hsp90 inhibitor synergizes with anti-PD1/anti-CTLA4 checkpoint inhibitor therapy in mice and results in a dramatic reduction in tumour size with a concomitant increase in survival (Rahmy et al., 2022). However, disruption of cellular proteostasis has the potential to alter the immunological features of tumour cells as well as the properties of immune cells (Mercier and LaPointe, 2022). We have shown that Hsp90 inhibition upregulates surface MHC1 across several cancer cell types. This increase in surface MHC1 presentation occurs in response to inhibition by all of the Hsp90 inhibitors we tested. This suggests that only inhibition of the cytosolic Hsp90 paralogues, Hsp90ɑ and Hsp90β, is required for the effect. Our data also suggest that inhibition of the ER Hsp90, Grp94, does not prevent MHC1 biogenesis and trafficking to the cell surface. We also show that the upregulation of MHC1 is specific for certain HLA-subtypes. If increased protein turnover and a concomitant increase in the number of peptides entering the ER for loading was driving the upregulation of surface MHC1 then one would expect to see an increase across all subtypes. However, that the increase is limited to HLA-B and HLA-C suggests that there is some mechanism for regulating what subtype is loaded and mobilized to the surface under these conditions. More work is required to determine if HLA-B and HLA-C play a role in modulating immune responses under conditions of proteotoxic stress in a way that HLA-A does not.

We observed that Hsp90 inhibition also increases surface PD-L1, which has anti-immune effects within the tumour microenvironment (Akinleye and Rasool, 2019; Liu et al., 2021). Previous work with the MC38 syngeneic mouse model shows that Hsp90 inhibitor treatment results in a decrease in tumour volume (Jaeger et al., 2019; Zavareh et al., 2021; Rahmy et al., 2022) as well as a decrease in surface levels of PD-L1 (Zavareh et al., 2021). Work in human THP-1 monocytic cells (where both mRNA and protein levels of PD-L1 were reduced with Hsp90 inhibitor treatment) suggested that this could be due to impaired transcription factor function (i.e., c-myc and Stat3) (Zavareh et al., 2021) but in the context of the tumour microenvironemnt, this could be due to the selection of PD-L1-independent tumour cell populations. We also observed that Hsp90 inhibition resulted in an increase in IFNGR surface levels but also blocked IFN-γ signaling which is an important consideration for future studies. Nonetheless, there is still significant potential for the use of proteostasis disrupters such as an Hsp90 inhibitor as a cancer therapeutic. Upregulation of PD-L1 can be overcome by immune checkpoint blockade therapy to neutralize the PD-1/PD-L1 interaction. Therefore, the combination of an Hsp90 inhibitor and ICIT checkpoint therapy has the potential to be an effective treatment for cancer. Future work can now be focused on examining the other effects of Hsp90 inhibition on the immune system and tumour microenvironment, and how other disruptions of proteostasis alter cancer immunogenicity.

## Data Availability

The raw data supporting the conclusion of this article will be made available by the authors, without undue reservation.

## Supplementary material

SUPPLEMENTARY FIGURE S1 Inhibition of Hsp90 in MDA-MB-231 cells using NVP-AUY922 results in a fold decrease in EGFR density. Cells were treated with 1µM of inhibitor for 24 hours. The abundance of EGFR on the cell surface was measured by flow cytometry using a EGFR antibody conjugated to PE and expressed as a fold-change over vehicle-treated cells. Experiments were carried out three times in triplicate (n=3) and statistical significance was determined using a t-test comparing the mean between each treatment group and the control group (* denotes p < 0.5; ** denotes p < 0.01; *** denotes p < 0.001; **** denotes p < 0.0001). Error bars show standard error of the mean.

SUPPLEMENTARY FIGURE S2 Raw data of cells treated with either DMSO **(A)** or 10µM NVP **(B)** and analyzed via Flow cytometry to demonstrate that cells were gated to exclude dead cells/debris (top left) and doublets (top right).
