# Supplementary figures and images for: Hsp90 inhibition leads to an increase in surface expression of multiple immunological receptors in cancer cells
Source: Front Mol Biosci. 2024 Apr 5;11:1334876. doi: 10.3389/fmolb.2024.1334876 (PMC11027010; doi:10.3389/fmolb.2024.1334876)

# Supplementary Figure 1

**Fold Change in EGFR of  
MDA-MB-231 Cells**

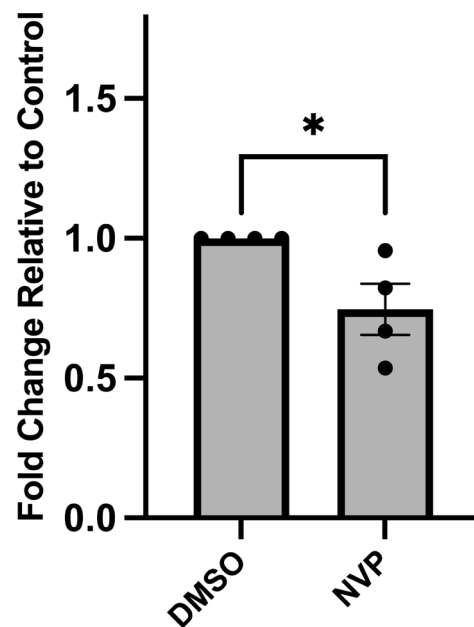

# Supplementary Figure 2

A

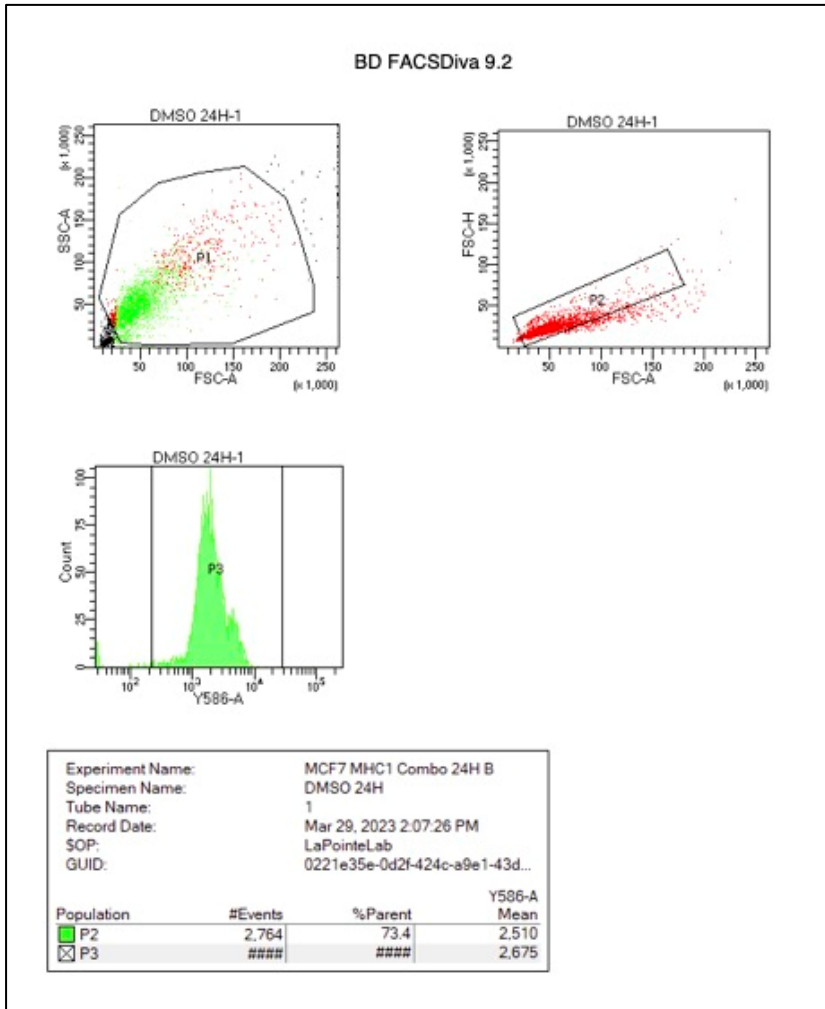

B

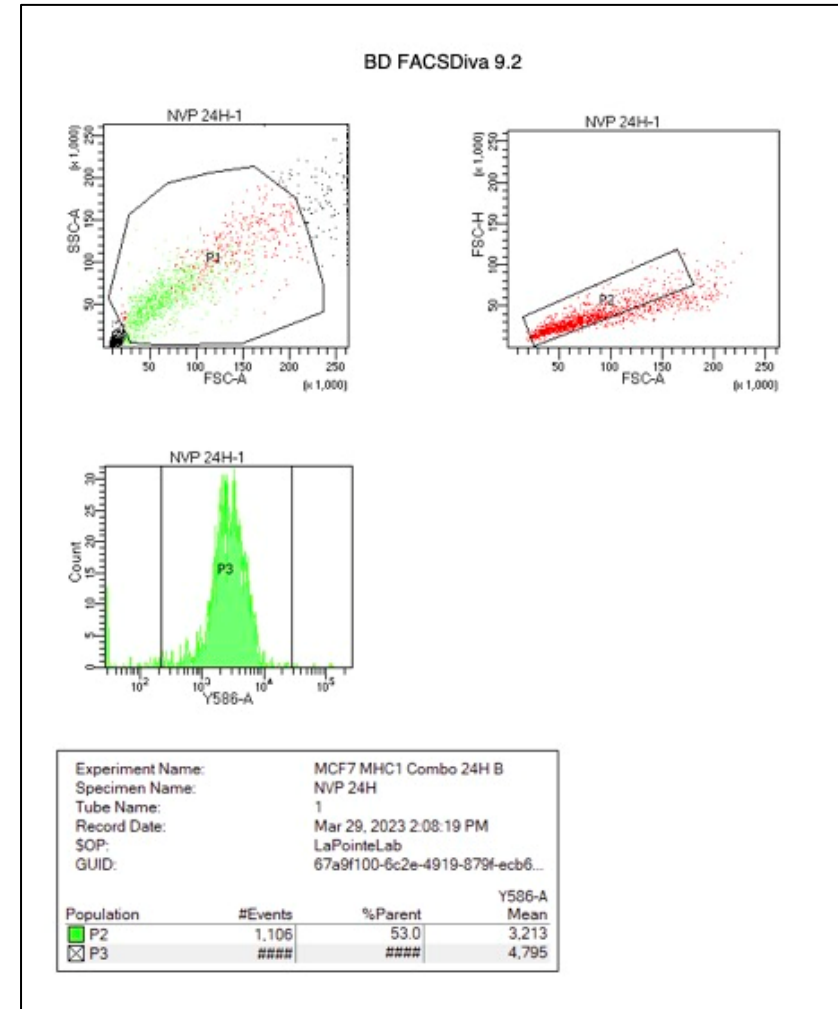

Supplement: Supplementary file 1 [file Image1.pdf]
